# 3,3′-Dicyclo­pentyl-1,1′-(1,3-phenyl­enedimethyl­ene)dibenzimidazol-1-ium bis­(hexa­fluoro­phosphate)

**DOI:** 10.1107/S160053681202274X

**Published:** 2012-05-23

**Authors:** Rosenani A. Haque, S. Fatimah Nasri, Mohd Mustaqim Rosli, Hoong-Kun Fun

**Affiliations:** a School of Chemical Sciences, Universiti Sains Malaysia, 11800 USM, Penang, Malaysia; b X-ray Crystallography Unit, School of Physics, Universiti Sains Malaysia, 11800 USM, Penang, Malaysia

## Abstract

In the title compound, C_32_H_36_N_4_^2+^·2PF_6_^−^, the cation and the anions each have crystallographic twofold rotation symmetry. The benzimidazole ring is almost planar [r.m.s. deviation = 0.0161 (1) Å] and makes a dihedral angle of 5.77 (4)° with its symmetry-related component and a dihedral angle of 80.96 (5)° with the central benzene ring. The cyclo­pentyl ring adopts a half-chair conformation. In the crystal, mol­ecules are linked into a three-dimensional network through C—H⋯F hydrogen bonds. A C—H⋯π inter­action is also observed.

## Related literature

For the biological activity of benzimidazole, see: Shaharyar *et al.* (2012 ▶); Mohan *et al.* (2011 ▶). For related structures, see: Haque *et al.* (2011 ▶, 2012 ▶). For puckering parameters, see: Cremer & Pople (1975 ▶).
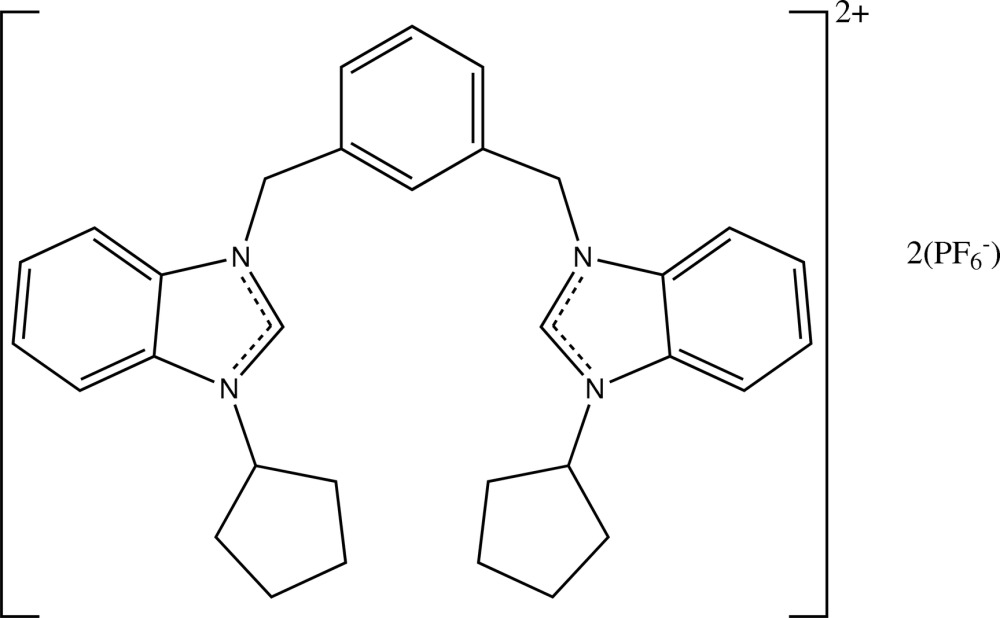


## Experimental

### 

#### Crystal data

C_32_H_36_N_4_^2+^·2PF_6_^−^
*M* *_r_* = 766.59
Orthorhombic, 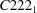

*a* = 7.0699 (1) Å
*b* = 20.4852 (3) Å
*c* = 22.5416 (3) Å
*V* = 3264.66 (8) Å^3^
*Z* = 4
Mo *K*α radiation
μ = 0.23 mm^−1^
*T* = 100 K
0.44 × 0.13 × 0.11 mm

#### Data collection

Bruker SMART APEXII CCD area-detector diffractometer
Absorption correction: multi-scan (*SADABS*; Bruker, 2009 ▶) *T*_min_ = 0.903, *T*_max_ = 0.974
19037 measured reflections
4758 independent reflections
4407 reflections with *I* > 2σ(*I*)
*R*_int_ = 0.024

#### Refinement

*R*[*F* ^2^ > 2σ(*F* ^2^)] = 0.032
*wR*(*F* ^2^) = 0.078
*S* = 1.05
4758 reflections
228 parameters
H-atom parameters constrained
Δρ_max_ = 0.29 e Å^−3^
Δρ_min_ = −0.24 e Å^−3^
Absolute structure: Flack (1983 ▶), 2086 Friedel pairs
Flack parameter: 0.02 (7)

### 

Data collection: *APEX2* (Bruker, 2009 ▶); cell refinement: *SAINT* (Bruker, 2009 ▶); data reduction: *SAINT*; program(s) used to solve structure: *SHELXTL* (Sheldrick, 2008 ▶); program(s) used to refine structure: *SHELXTL*; molecular graphics: *SHELXTL*; software used to prepare material for publication: *SHELXTL* and *PLATON* (Spek, 2009 ▶).

## Supplementary Material

Crystal structure: contains datablock(s) I, global. DOI: 10.1107/S160053681202274X/rz2756sup1.cif

Structure factors: contains datablock(s) I. DOI: 10.1107/S160053681202274X/rz2756Isup2.hkl

Supplementary material file. DOI: 10.1107/S160053681202274X/rz2756Isup3.cml

Additional supplementary materials:  crystallographic information; 3D view; checkCIF report

## Figures and Tables

**Table 1:** Hydrogen-bond geometry (Å, °)

*D*—H⋯*A*	*D*—H	H⋯*A*	*D*⋯*A*	*D*—H⋯*A*
C2—H2*A*⋯F3^i^	0.95	2.44	3.3814 (18)	171
C5—H5*B*⋯F4^ii^	0.99	2.41	3.2670 (17)	145
C8—H8*A*⋯F2^iii^	0.95	2.46	3.3912 (18)	167
C12—H12*A*⋯F4^ii^	0.95	2.42	3.2252 (17)	142
C13—H13*A*⋯F2^iv^	1.00	2.51	3.4090 (18)	150
C13—H13*A*⋯F3^iv^	1.00	2.31	3.2124 (17)	149
C9—H9*A*⋯*Cg*1^iv^	0.95	2.79	3.6416 (15)	149

Symmetry codes: (i) 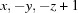; (ii) 
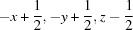
; (iii) 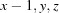; (iv) 
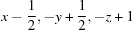
.

**Fig. 1.**
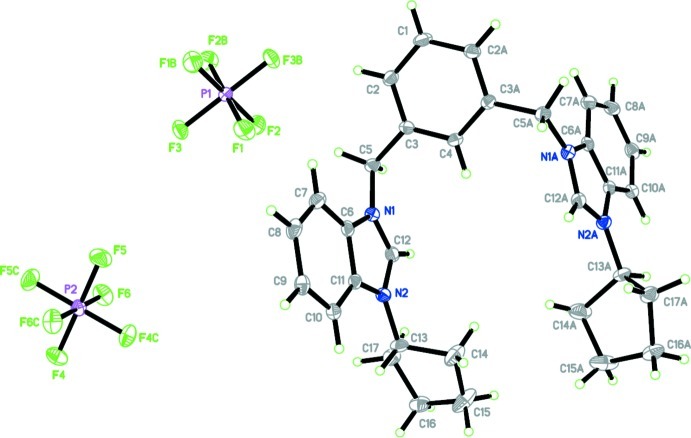
The molecular structure of the title compound, showing 50% probability displacement ellipsoids. Hydrogen atoms are shown as spheres of arbitrary radius. Atoms with suffix A, B and C are generated by the symmetry operators (-x, y, 1/2-z), (x, -y, 1-z) and (-x, y, 3/2-z), respectively.

**Fig. 2.**
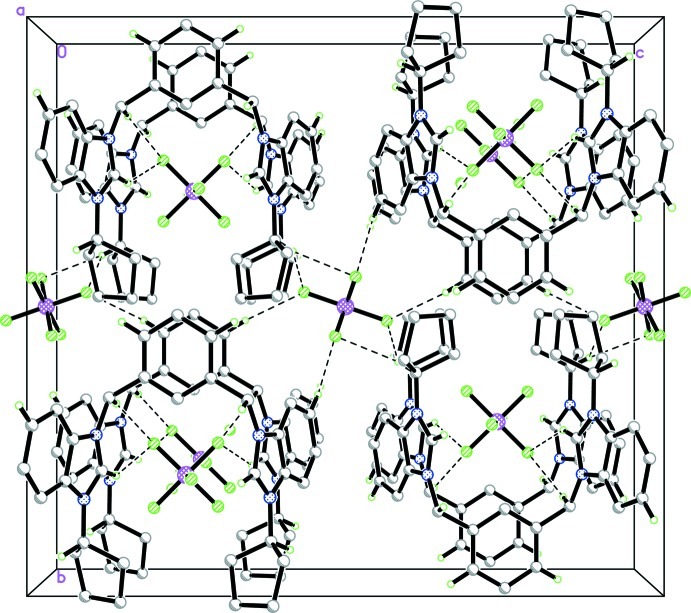
The crystal packing of the title compound. Dashed lines indicate hydrogen bonds. H atoms not involved in hydrogen bonding interactions have been omitted for clarity.

## supplementary crystallographic information

### Comment

Benzimidazole, a heterocyclic aromatic organic compound, has a wide variety of biological activities (Shaharyar *et al.*, 2012; Mohan *et al.*, 2011). Previously, we have reported the crystal structures of benzimidazole with various substitutions (Haque *et al.*, 2011, 2012). In this report, we describe the crystal structure of a *meta*-xylyl linked bis-benzimidazolium salt with cyclopentyl substitution.

All parameters in the title compound (Fig. 1) are within normal ranges. The complete molecule, as well as the anions, is generated by a crystallographic twofold axis. The benzimidazole (N1—N2/C6—C12) ring is planar with the r.m.s. 0.0161 (1) Å. It makes a dihedral angle of 5.77 (4)° with its symmetry-related component and of 80.96 (5)° with the central benzene ring (C1—C4/C2A—C3A). The cyclopentyl ring adopts a half chair conformation with puckering parameters *Q* = 0.4256 (18) Å and φ = 344.9 (3)° (Cremer & Pople, 1975).

In the crystal structure (Fig. 2), the molecules are linked into a three-dimensional network through intermolecular C—H···F hydrogen bonds and C—H···π interactions involving the centroid of the C6—C11 ring (Table 1).

### Experimental

To a solution of 1,3-(bromomethyl)benzene (2.64 g, 0.01 mol) in 50 ml of 1,4-dioxane, 1-cyclopentyl-1*H*-benzolimidazole (3.73 g, 0.02 mol) was added. The mixture was refluxed at 373 K for 24 h. The resulting brown thick liquid product was decanted, washed with fresh 1,4-dioxane (3 × 5 ml) and converted directly to its hexafluorophosphate counterpart by metathesis reaction using KPF_6_ (3.60 g, 0.02 mol) in 40 ml of methanol/water. The white precipitate was collected, washed with fresh methanol to give the title product as a white solid (4.66 g, 97%). M.p. 518–519 K. Crystals suitable for X-ray diffraction studies were obtained by slow evaporation of the salt solution in a mixture of acetonitrile/methanol (1:1 *v*/*v*) at ambient temperature.

### Refinement

All H atoms attached to C atoms were fixed geometrically and refined as riding with C—H = 0.95–1.00 Å and *U*_iso_(H) = 1.2*U*_eq_(C).

### Crystal data

**Table:**

C_32_H_36_N_4_^2+^·2PF_6_^−^	*F*(000) = 1576
*M**_r_* = 766.59	*D*_x_ = 1.560 Mg m^−^^3^
Orthorhombic, *C*222_1_	Mo *K*α radiation, λ = 0.71073 Å
Hall symbol: C 2c 2	Cell parameters from 9943 reflections
*a* = 7.0699 (1) Å	θ = 2.2–29.9°
*b* = 20.4852 (3) Å	µ = 0.23 mm^−^^1^
*c* = 22.5416 (3) Å	*T* = 100 K
*V* = 3264.66 (8) Å^3^	Block, colourless
*Z* = 4	0.44 × 0.13 × 0.11 mm

### Data collection

**Table:**

Bruker SMART APEXII CCD area-detector diffractometer	4758 independent reflections
Radiation source: fine-focus sealed tube	4407 reflections with *I* > 2σ(*I*)
Graphite monochromator	*R*_int_ = 0.024
φ and ω scans	θ_max_ = 30.0°, θ_min_ = 1.8°
Absorption correction: multi-scan (*SADABS*; Bruker, 2009)	*h* = −9→9
*T*_min_ = 0.903, *T*_max_ = 0.974	*k* = −28→28
19037 measured reflections	*l* = −28→31

### Refinement

**Table:**

Refinement on *F*^2^	Secondary atom site location: difference Fourier map
Least-squares matrix: full	Hydrogen site location: inferred from neighbouring sites
*R*[*F*^2^ > 2σ(*F*^2^)] = 0.032	H-atom parameters constrained
*wR*(*F*^2^) = 0.078	*w* = 1/[σ^2^(*F*_o_^2^) + (0.0411*P*)^2^ + 1.0871*P*] where *P* = (*F*_o_^2^ + 2*F*_c_^2^)/3
*S* = 1.05	(Δ/σ)_max_ = 0.001
4758 reflections	Δρ_max_ = 0.29 e Å^−^^3^
228 parameters	Δρ_min_ = −0.24 e Å^−^^3^
0 restraints	Absolute structure: Flack (1983), 2086 Friedel pairs
Primary atom site location: structure-invariant direct methods	Flack parameter: 0.02 (7)

### Special details

**Table:**

Experimental. The crystal was placed in the cold stream of an Oxford Cryosystems Cobra open-flow nitrogen cryostat (Cosier & Glazer, 1986) operating at 100.0 (1) K.
Geometry. All e.s.d.'s (except the e.s.d. in the dihedral angle between two l.s. planes) are estimated using the full covariance matrix. The cell e.s.d.'s are taken into account individually in the estimation of e.s.d.'s in distances, angles and torsion angles; correlations between e.s.d.'s in cell parameters are only used when they are defined by crystal symmetry. An approximate (isotropic) treatment of cell e.s.d.'s is used for estimating e.s.d.'s involving l.s. planes.
Refinement. Refinement of *F*^2^ against ALL reflections. The weighted *R*-factor *wR* and goodness of fit *S* are based on *F*^2^, conventional *R*-factors *R* are based on *F*, with *F* set to zero for negative *F*^2^. The threshold expression of *F*^2^ > σ(*F*^2^) is used only for calculating *R*-factors(gt) *etc*. and is not relevant to the choice of reflections for refinement. *R*-factors based on *F*^2^ are statistically about twice as large as those based on *F*, and *R*- factors based on ALL data will be even larger.

### Fractional atomic coordinates and isotropic or equivalent isotropic displacement parameters (Å^2^)

**Table:**

	*x*	*y*	*z*	*U*_iso_*/*U*_eq_	
P1	0.12643 (8)	0.0000	0.5000	0.01579 (10)	
F1	−0.03216 (16)	0.05234 (5)	0.48352 (4)	0.0333 (2)	
F2	0.28761 (15)	0.05230 (5)	0.48369 (4)	0.0313 (2)	
F3	0.12649 (14)	0.02573 (5)	0.56726 (4)	0.02558 (19)	
P2	0.0000	0.20928 (3)	0.7500	0.01858 (11)	
F4	0.03085 (15)	0.26421 (5)	0.79953 (4)	0.0304 (2)	
F5	−0.03160 (16)	0.15392 (5)	0.70063 (4)	0.0331 (2)	
F6	0.22344 (14)	0.20932 (5)	0.73648 (4)	0.0307 (2)	
N1	0.01439 (18)	0.21030 (6)	0.37157 (5)	0.0166 (2)	
N2	−0.04694 (17)	0.31380 (6)	0.38710 (5)	0.0158 (2)	
C1	0.0000	0.01437 (9)	0.2500	0.0217 (4)	
H1A	0.0000	−0.0320	0.2500	0.026*	
C2	0.0497 (2)	0.04841 (7)	0.30090 (7)	0.0187 (3)	
H2A	0.0830	0.0252	0.3359	0.022*	
C3	0.0511 (2)	0.11646 (6)	0.30090 (6)	0.0155 (3)	
C4	0.0000	0.15040 (9)	0.2500	0.0171 (4)	
H4A	0.0000	0.1968	0.2500	0.020*	
C5	0.1218 (2)	0.15116 (6)	0.35629 (6)	0.0184 (3)	
H5A	0.1158	0.1204	0.3901	0.022*	
H5B	0.2561	0.1632	0.3504	0.022*	
C6	−0.1642 (2)	0.21287 (7)	0.39812 (6)	0.0159 (3)	
C7	−0.2882 (2)	0.16395 (7)	0.41598 (7)	0.0203 (3)	
H7A	−0.2614	0.1191	0.4095	0.024*	
C8	−0.4523 (2)	0.18423 (7)	0.44364 (7)	0.0215 (3)	
H8A	−0.5404	0.1524	0.4568	0.026*	
C9	−0.4927 (2)	0.25100 (7)	0.45287 (6)	0.0194 (3)	
H9A	−0.6077	0.2628	0.4718	0.023*	
C10	−0.3694 (2)	0.29965 (7)	0.43506 (6)	0.0168 (3)	
H10A	−0.3966	0.3446	0.4411	0.020*	
C11	−0.2030 (2)	0.27901 (6)	0.40778 (6)	0.0157 (3)	
C12	0.0780 (2)	0.27128 (7)	0.36572 (6)	0.0173 (3)	
H12A	0.1962	0.2828	0.3486	0.021*	
C13	−0.0262 (2)	0.38548 (6)	0.38878 (7)	0.0187 (3)	
H13A	−0.1242	0.4034	0.4163	0.022*	
C14	−0.0516 (3)	0.41777 (8)	0.32852 (8)	0.0328 (4)	
H14A	−0.1872	0.4213	0.3181	0.039*	
H14B	0.0147	0.3929	0.2971	0.039*	
C15	0.0376 (3)	0.48588 (8)	0.33678 (10)	0.0377 (5)	
H15A	0.1187	0.4970	0.3024	0.045*	
H15B	−0.0620	0.5196	0.3407	0.045*	
C16	0.1564 (2)	0.48203 (7)	0.39385 (8)	0.0258 (3)	
H16A	0.2848	0.4998	0.3871	0.031*	
H16B	0.0956	0.5070	0.4262	0.031*	
C17	0.1659 (2)	0.40913 (7)	0.40946 (7)	0.0233 (3)	
H17A	0.2696	0.3870	0.3879	0.028*	
H17B	0.1828	0.4024	0.4526	0.028*	

### Atomic displacement parameters (Å^2^)

**Table:**

	*U*^11^	*U*^22^	*U*^33^	*U*^12^	*U*^13^	*U*^23^
P1	0.0180 (2)	0.0139 (2)	0.0155 (2)	0.000	0.000	0.00104 (18)
F1	0.0360 (6)	0.0354 (5)	0.0287 (5)	0.0206 (4)	−0.0024 (4)	−0.0028 (4)
F2	0.0348 (6)	0.0286 (5)	0.0304 (5)	−0.0119 (4)	0.0112 (4)	0.0012 (4)
F3	0.0298 (5)	0.0311 (4)	0.0158 (4)	−0.0089 (4)	0.0008 (4)	−0.0032 (4)
P2	0.0195 (3)	0.0204 (2)	0.0159 (2)	0.000	−0.0036 (2)	0.000
F4	0.0330 (6)	0.0316 (5)	0.0266 (5)	0.0000 (4)	−0.0041 (4)	−0.0115 (4)
F5	0.0414 (6)	0.0304 (5)	0.0276 (5)	−0.0014 (5)	−0.0082 (5)	−0.0105 (4)
F6	0.0202 (5)	0.0419 (5)	0.0300 (6)	0.0038 (4)	−0.0009 (4)	−0.0035 (4)
N1	0.0196 (6)	0.0151 (5)	0.0152 (5)	−0.0003 (5)	0.0004 (5)	−0.0011 (4)
N2	0.0162 (6)	0.0144 (5)	0.0168 (6)	−0.0026 (4)	0.0024 (4)	−0.0011 (4)
C1	0.0268 (11)	0.0110 (8)	0.0273 (11)	0.000	−0.0015 (9)	0.000
C2	0.0206 (7)	0.0158 (6)	0.0197 (7)	0.0016 (5)	0.0000 (6)	0.0027 (5)
C3	0.0161 (6)	0.0135 (6)	0.0169 (6)	0.0001 (5)	0.0011 (5)	−0.0018 (5)
C4	0.0208 (9)	0.0116 (8)	0.0188 (9)	0.000	0.0011 (8)	0.000
C5	0.0219 (7)	0.0146 (6)	0.0186 (7)	0.0048 (6)	−0.0030 (6)	−0.0007 (5)
C6	0.0186 (7)	0.0180 (6)	0.0111 (6)	−0.0010 (5)	−0.0022 (5)	0.0007 (5)
C7	0.0241 (8)	0.0165 (6)	0.0202 (7)	−0.0031 (5)	−0.0041 (6)	0.0024 (5)
C8	0.0221 (7)	0.0226 (7)	0.0199 (7)	−0.0079 (6)	−0.0005 (6)	0.0058 (6)
C9	0.0168 (7)	0.0252 (7)	0.0163 (6)	−0.0033 (6)	0.0004 (6)	0.0029 (5)
C10	0.0183 (6)	0.0175 (6)	0.0145 (6)	−0.0002 (5)	0.0002 (5)	0.0004 (5)
C11	0.0194 (7)	0.0156 (6)	0.0121 (6)	−0.0036 (5)	−0.0014 (5)	0.0002 (5)
C12	0.0190 (7)	0.0182 (6)	0.0149 (6)	−0.0012 (5)	0.0012 (5)	−0.0004 (5)
C13	0.0191 (7)	0.0116 (5)	0.0253 (7)	−0.0020 (5)	0.0043 (6)	−0.0021 (5)
C14	0.0383 (10)	0.0226 (8)	0.0376 (10)	−0.0044 (7)	−0.0151 (8)	0.0105 (7)
C15	0.0298 (9)	0.0254 (8)	0.0578 (13)	−0.0078 (7)	−0.0142 (9)	0.0182 (8)
C16	0.0223 (8)	0.0176 (7)	0.0376 (9)	−0.0050 (5)	0.0037 (7)	−0.0022 (6)
C17	0.0241 (8)	0.0192 (7)	0.0267 (8)	−0.0060 (6)	−0.0051 (6)	−0.0001 (6)

### Geometric parameters (Å, º)

**Table:**

P1—F1	1.5951 (10)	C5—H5A	0.9900
P1—F1^i^	1.5952 (10)	C5—H5B	0.9900
P1—F3	1.6051 (9)	C6—C7	1.391 (2)
P1—F3^i^	1.6051 (9)	C6—C11	1.3993 (19)
P1—F2	1.6067 (10)	C7—C8	1.381 (2)
P1—F2^i^	1.6067 (10)	C7—H7A	0.9500
P2—F4^ii^	1.6002 (10)	C8—C9	1.413 (2)
P2—F4	1.6002 (10)	C8—H8A	0.9500
P2—F5	1.6046 (10)	C9—C10	1.384 (2)
P2—F5^ii^	1.6046 (10)	C9—H9A	0.9500
P2—F6	1.6088 (10)	C10—C11	1.393 (2)
P2—F6^ii^	1.6088 (10)	C10—H10A	0.9500
N1—C12	1.3342 (18)	C12—H12A	0.9500
N1—C6	1.3981 (19)	C13—C17	1.515 (2)
N1—C5	1.4707 (17)	C13—C14	1.522 (2)
N2—C12	1.3307 (18)	C13—H13A	1.0000
N2—C11	1.3935 (18)	C14—C15	1.542 (2)
N2—C13	1.4762 (17)	C14—H14A	0.9900
C1—C2^iii^	1.3879 (18)	C14—H14B	0.9900
C1—C2	1.3879 (18)	C15—C16	1.538 (3)
C1—H1A	0.9500	C15—H15A	0.9900
C2—C3	1.3942 (19)	C15—H15B	0.9900
C2—H2A	0.9500	C16—C17	1.536 (2)
C3—C4	1.3894 (17)	C16—H16A	0.9900
C3—C5	1.5210 (19)	C16—H16B	0.9900
C4—C3^iii^	1.3894 (17)	C17—H17A	0.9900
C4—H4A	0.9500	C17—H17B	0.9900
			
F1—P1—F1^i^	90.68 (9)	H5A—C5—H5B	107.6
F1—P1—F3	89.97 (5)	C7—C6—N1	131.74 (13)
F1^i^—P1—F3	90.05 (5)	C7—C6—C11	121.94 (14)
F1—P1—F3^i^	90.05 (5)	N1—C6—C11	106.27 (12)
F1^i^—P1—F3^i^	89.97 (5)	C8—C7—C6	116.32 (13)
F3—P1—F3^i^	179.97 (8)	C8—C7—H7A	121.8
F1—P1—F2	89.83 (6)	C6—C7—H7A	121.8
F1^i^—P1—F2	179.47 (7)	C7—C8—C9	121.84 (14)
F3—P1—F2	89.83 (5)	C7—C8—H8A	119.1
F3^i^—P1—F2	90.15 (5)	C9—C8—H8A	119.1
F1—P1—F2^i^	179.47 (7)	C10—C9—C8	121.82 (14)
F1^i^—P1—F2^i^	89.83 (6)	C10—C9—H9A	119.1
F3—P1—F2^i^	90.15 (5)	C8—C9—H9A	119.1
F3^i^—P1—F2^i^	89.83 (5)	C9—C10—C11	116.20 (12)
F2—P1—F2^i^	89.66 (8)	C9—C10—H10A	121.9
F4^ii^—P2—F4	90.62 (8)	C11—C10—H10A	121.9
F4^ii^—P2—F5	89.66 (5)	C10—C11—N2	131.36 (12)
F4—P2—F5	179.64 (6)	C10—C11—C6	121.87 (13)
F4^ii^—P2—F5^ii^	179.64 (6)	N2—C11—C6	106.74 (12)
F4—P2—F5^ii^	89.66 (5)	N2—C12—N1	110.70 (13)
F5—P2—F5^ii^	90.05 (8)	N2—C12—H12A	124.6
F4^ii^—P2—F6	90.07 (6)	N1—C12—H12A	124.6
F4—P2—F6	89.89 (6)	N2—C13—C17	114.52 (12)
F5—P2—F6	90.33 (6)	N2—C13—C14	113.44 (12)
F5^ii^—P2—F6	89.72 (6)	C17—C13—C14	103.97 (13)
F4^ii^—P2—F6^ii^	89.88 (6)	N2—C13—H13A	108.2
F4—P2—F6^ii^	90.07 (6)	C17—C13—H13A	108.2
F5—P2—F6^ii^	89.71 (6)	C14—C13—H13A	108.2
F5^ii^—P2—F6^ii^	90.33 (6)	C13—C14—C15	103.71 (14)
F6—P2—F6^ii^	179.94 (9)	C13—C14—H14A	111.0
C12—N1—C6	108.13 (12)	C15—C14—H14A	111.0
C12—N1—C5	125.04 (13)	C13—C14—H14B	111.0
C6—N1—C5	126.70 (12)	C15—C14—H14B	111.0
C12—N2—C11	108.16 (11)	H14A—C14—H14B	109.0
C12—N2—C13	126.47 (12)	C16—C15—C14	106.14 (13)
C11—N2—C13	125.37 (12)	C16—C15—H15A	110.5
C2^iii^—C1—C2	119.68 (17)	C14—C15—H15A	110.5
C2^iii^—C1—H1A	120.2	C16—C15—H15B	110.5
C2—C1—H1A	120.2	C14—C15—H15B	110.5
C1—C2—C3	120.28 (14)	H15A—C15—H15B	108.7
C1—C2—H2A	119.9	C17—C16—C15	105.39 (13)
C3—C2—H2A	119.9	C17—C16—H16A	110.7
C4—C3—C2	119.91 (13)	C15—C16—H16A	110.7
C4—C3—C5	121.97 (12)	C17—C16—H16B	110.7
C2—C3—C5	118.01 (12)	C15—C16—H16B	110.7
C3^iii^—C4—C3	119.95 (17)	H16A—C16—H16B	108.8
C3^iii^—C4—H4A	120.0	C13—C17—C16	101.60 (12)
C3—C4—H4A	120.0	C13—C17—H17A	111.5
N1—C5—C3	114.05 (12)	C16—C17—H17A	111.5
N1—C5—H5A	108.7	C13—C17—H17B	111.5
C3—C5—H5A	108.7	C16—C17—H17B	111.5
N1—C5—H5B	108.7	H17A—C17—H17B	109.3
C3—C5—H5B	108.7		
			
C2^iii^—C1—C2—C3	−0.43 (10)	C12—N2—C11—C6	0.54 (16)
C1—C2—C3—C4	0.9 (2)	C13—N2—C11—C6	−179.62 (13)
C1—C2—C3—C5	−175.19 (12)	C7—C6—C11—C10	−1.0 (2)
C2—C3—C4—C3^iii^	−0.43 (10)	N1—C6—C11—C10	−178.56 (12)
C5—C3—C4—C3^iii^	175.47 (15)	C7—C6—C11—N2	177.31 (13)
C12—N1—C5—C3	−108.17 (16)	N1—C6—C11—N2	−0.29 (15)
C6—N1—C5—C3	76.47 (17)	C11—N2—C12—N1	−0.60 (17)
C4—C3—C5—N1	42.77 (18)	C13—N2—C12—N1	179.57 (13)
C2—C3—C5—N1	−141.25 (14)	C6—N1—C12—N2	0.41 (16)
C12—N1—C6—C7	−177.33 (15)	C5—N1—C12—N2	−175.68 (13)
C5—N1—C6—C7	−1.3 (2)	C12—N2—C13—C17	−44.6 (2)
C12—N1—C6—C11	−0.06 (15)	C11—N2—C13—C17	135.57 (14)
C5—N1—C6—C11	175.94 (13)	C12—N2—C13—C14	74.5 (2)
N1—C6—C7—C8	177.12 (14)	C11—N2—C13—C14	−105.29 (17)
C11—C6—C7—C8	0.2 (2)	N2—C13—C14—C15	−162.32 (14)
C6—C7—C8—C9	0.5 (2)	C17—C13—C14—C15	−37.29 (18)
C7—C8—C9—C10	−0.5 (2)	C13—C14—C15—C16	15.5 (2)
C8—C9—C10—C11	−0.3 (2)	C14—C15—C16—C17	11.4 (2)
C9—C10—C11—N2	−176.85 (14)	N2—C13—C17—C16	168.57 (13)
C9—C10—C11—C6	1.0 (2)	C14—C13—C17—C16	44.24 (15)
C12—N2—C11—C10	178.59 (15)	C15—C16—C17—C13	−33.95 (17)
C13—N2—C11—C10	−1.6 (2)		

Symmetry codes: (i) *x*, −*y*, −*z*+1; (ii) −*x*, *y*, −*z*+3/2; (iii) −*x*, *y*, −*z*+1/2.

### Hydrogen-bond geometry (Å, º)

**Table:**

*D*—H···*A*	*D*—H	H···*A*	*D*···*A*	*D*—H···*A*
C2—H2*A*···F3^i^	0.95	2.44	3.3814 (18)	171
C5—H5*B*···F4^iv^	0.99	2.41	3.2670 (17)	145
C8—H8*A*···F2^v^	0.95	2.46	3.3912 (18)	167
C12—H12*A*···F4^iv^	0.95	2.42	3.2252 (17)	142
C13—H13*A*···F2^vi^	1.00	2.51	3.4090 (18)	150
C13—H13*A*···F3^vi^	1.00	2.31	3.2124 (17)	149
C9—H9*A*···*Cg*1^vi^	0.95	2.79	3.6416 (15)	149

Symmetry codes: (i) *x*, −*y*, −*z*+1; (iv) −*x*+1/2, −*y*+1/2, *z*−1/2; (v) *x*−1, *y*, *z*; (vi) *x*−1/2, −*y*+1/2, −*z*+1.
